# Clinical calculator based on clinicopathological characteristics predicts local recurrence and overall survival following radical resection of stage II-III colorectal cancer

**DOI:** 10.3389/fonc.2025.1494255

**Published:** 2025-02-05

**Authors:** Fei Huang, Ran Wei, Shiwen Mei, Tixian Xiao, Wei Zhao, Zhaoxu Zheng, Qian Liu

**Affiliations:** ^1^ Department of Colorectal Surgery, National Cancer Center/National Clinical Research Center for Cancer/Cancer Hospital, Chinese Academy of Medical Sciences and Peking Union Medical College, Beijing, China; ^2^ Department of Gastrointestinal Surgery, The First Affiliated Hospital, Sun Yat-sen University, Guangzhou, Guangdong, China

**Keywords:** colorectal cancer, nomogram, clinical risk factor, prognosis, local recurrence

## Abstract

**Purpose:**

This study aimed to analyze the risk factors and survival prognosis of local recurrence in stage II-III colorectal cancer (CRC) and develop a clinical risk calculator and nomograms to predict local recurrence and survival in treated patients.

**Methods:**

Patients who underwent radical surgery between January 2009 and December 2019 at the China National Cancer Center were included. Multivariate nomograms and a clinical risk calculator based on Cox regression were developed. Discrimination was measured with an area under curve (AUC) and variability in individual predictions was assessed with calibration curves. We stratified patients into different risk groups according to the established model to predict their prognosis and guide clinical practice.

**Results:**

The clinical risk calculator incorporated six variables: tumor thrombus, perineural invasion, tumor grade, pathology T-stage, pathology N-stage, and whether more than 12 lymph nodes were harvested. Our clinical risk calculator provided good discrimination, with AUC values of local recurrence-free survival (LRFS) (0.764) and overall survival (OS) (0.815) in the training cohort and LRFS (0.740) and OS (0.730) in the test cohort. Calibration plots illustrated excellent agreement between the clinical risk calculator predictions and actual observations for 3- and 5-year LRFS and OS. Recurrence risk-stratified analysis showed that low-risk patients were more likely to undergo salvage radical surgery when recurrent disease existed.

**Conclusion:**

The clinical calculator can better account for tumor and patient heterogeneity, providing a more individualized outcome prognostication. The model is expected to aid in treatment planning, such as resectability evaluation, and it can be used in postoperative surveillance (https://oldcoloncancer.shinyapps.io/dynnomapp/).

## 1 Introduction

Whether the data are from the China National Cancer Center (CNCC) or the World Health Organization (WHO), colorectal cancer (CRC) is one of the most common cancers (1, 2). Although the widespread development of standardized surgical techniques [total mesorectal excision (TME) and complete mesocolic excision (CME)] and neoadjuvant chemoradiotherapy (CRT) in the past few decades has dramatically reduced the local recurrence rate (3), it still reaches 6% to 14% (4, 5) and is still an essential factor affecting the long-term survival of CRC patients. Compared with distant metastasis, it is easier to achieve disease control in locally recurrent disease and has more opportunities for surgery. However, clinically diagnosed locally recurrent CRC is often at an advanced stage of disease, and only approximately one-third of patients have resectable recurrent tumors (6–8). For those patients who can be treated with surgery, since recurrent tumors often have extensive infiltration, *en bloc* resection requires aggressive surgical strategies. If necessary, posterior pelvic excision (PPE) or even total pelvic excision (TPE) is needed. At the same time, the high complication rate, functional impairment, and perioperative death caused by surgery affect patients’ short-term and long-term prognoses (9, 10). Therefore, it is necessary to establish a prediction model for local recurrence of CRC to achieve early diagnosis and treatment.

Numerous studies have investigated the risk factors affecting recurrence after surgery and the ensuing overall survival rate (11). In addition, some studies have also developed postoperative prognostic models for CRC (12), but more research is needed in China. In recent years, nomograms have attracted increasing attention as tools that are easy to operate and powerful predictive statistical models. Since Henderson first reported the clinical application of nomograms (13), nomograms have been developed for various malignancies. They do not produce risk groups but attempt to combine all proven prognostic factors and quantify risk as precisely as possible (12).

In the present study, we collected data from patients with stage II or III CRC at the CNCC and focused on patients whose local recurrences were detected postoperatively. We first analyzed the factors that could affect the long-term survival of patients with locally recurrent CRC and then constructed a prognostic nomogram to predict the local recurrence-free survival (LRFS) and overall survival (OS) of these patients.

## 2 Materials and methods

### 2.1 Patients

**Figure 1:**
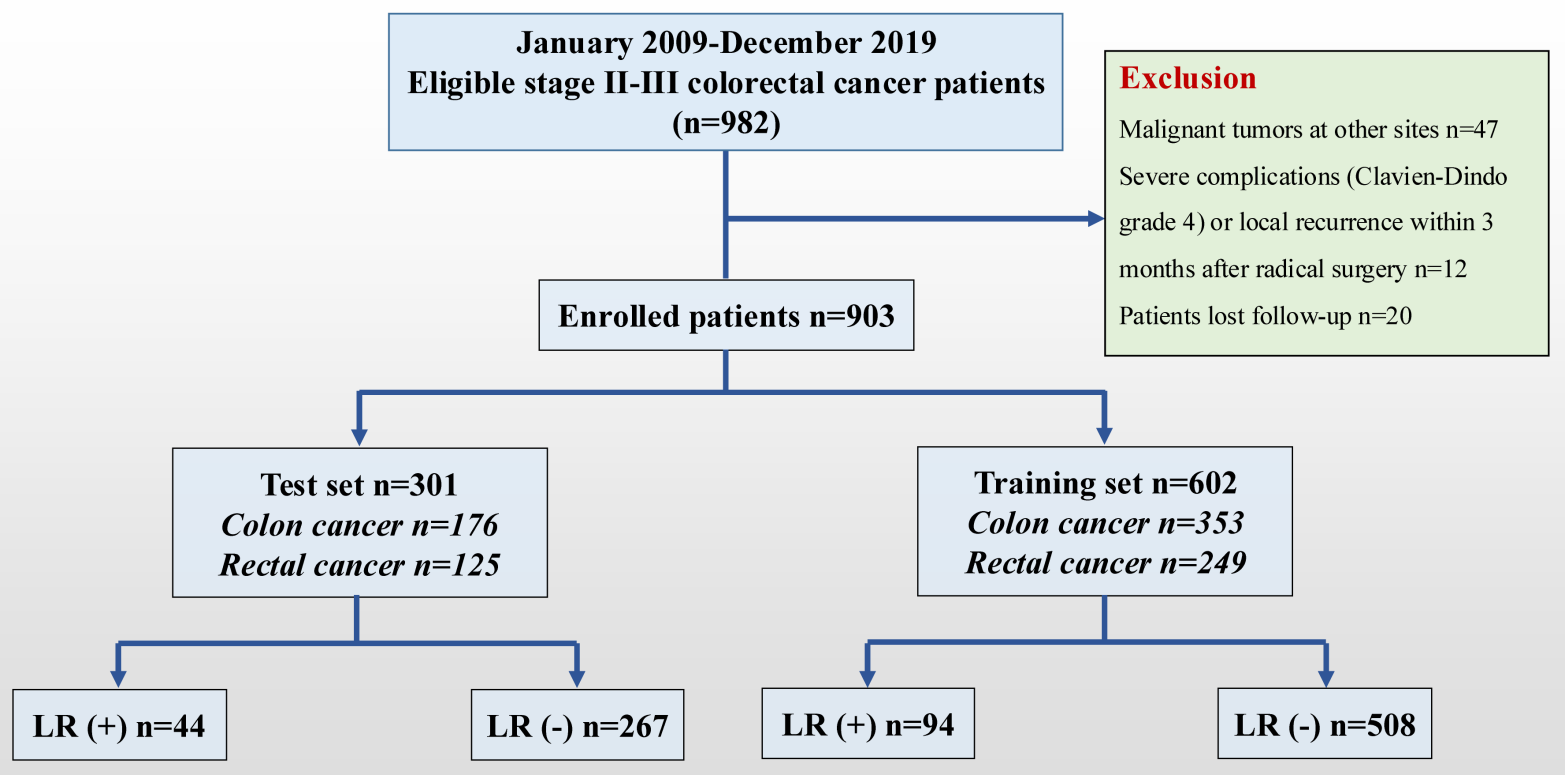
Flow diagram of the present study. CRC, colorectal cancer; LR, local recurrence.

A consecutive cohort study of patients with stages II-III CRC who underwent radical surgery at the CNCC was retrospectively conducted between January 2009 and December 2019. The tumor location was determined according to the surgical records. Of the 982 stage II-III patients in the database, 903 patients met the inclusion criteria (**Figure 1**). The tumor location was determined according to the surgical records and rectosigmoid carcinomas were classified as sigmoid carcinomas. The dominant (clinically more advanced) cancer data were collected if the patient presented simultaneously with multiple colon or rectal cancers. The inclusion criteria were as follows: (1) patients with stage II-III CRC who underwent radical surgery (R0 resection); (2) pathological type of adenocarcinoma (including mucinous adenocarcinoma and signet ring cell carcinoma); and (3) complete medical records and continuous follow-up records. The exclusion criteria were as follows: (1) familial hereditary rectal malignant tumor; (2) previous history of malignant tumor; and (3) severe complications (Clavien–Dindo grade 4) or local recurrence within 3 months after radical surgery. The CNCC Committee approved this study (20/355-2551).

### 2.2 Definition of locally recurrent CRC

One of the difficulties in assessing the outcomes of treatment for CRC in metachronous recurrences is that there is no consistent definition of the metachronous events in the published literature. We considered local recurrences as those diagnosed more than 3 months after radical surgery. In addition, locally recurrent CRC has been defined heterogeneously in the literature and between different institutions (14). Especially in China, surgeons often describe local recurrence as confined to the pelvic or abdominal cavity without distant metastasis, which makes it easier to analyze independent risk factors for long-term survival after local recurrence in patients. However, this study aimed to explore the factors that cause local recurrence so it defined local recurrence CRC as recurrence at the site around the tumor bed, the anastomotic site, or tissues around the intestine or mesenteric or regional lymph nodes after R0 surgery, with or without metastasis in the parenchymal organ. The diagnosis of local recurrence was confirmed by computed tomography (CT) scan, magnetic resonance imaging (MRI), positron emission tomography (PET-CT), or pathology (biopsy or specimen). According to different recurrence sites, locally recurrent colon cancer was categorized as peri-anastomotic, mesenteric/lymph nodal, peritoneal, retroperitoneal, abdominal wall, or pelvic (15). The location of the locally recurrent rectal cancer was categorized as central, sidewall, sacral, or mixed (16). More than one type of unifocal recurrence involved was categorized as multiple.

### 2.3 Follow-up

Outcomes of interest were factors influencing LRFS and OS. Starting with the first recurrence of surgical treatment, follow-ups will be conducted every 3 months for 2 years, then every 6 months, and annually after 5 years. Evaluation at follow-up included physical examination; colonoscopy; carcinoembryonic antigen (CEA) level; carbohydrate antigen 19-9 (CA199) level; chest, abdomen, and pelvis contrast-enhanced CT; and pelvic MRI or PET-CT as necessary. LRFS was defined as the time from radical surgery to the first local recurrence. OS was defined as the time from surgical treatment to death from any cause.

### 2.4 Statistical analysis

The patients from the CNCC were separated into training and test sets, which are shown in **Table 1**. The χ2 test was performed to compare differences in categorical variables (e.g., sex) in the composition ratios of the two groups, and the Wilcoxon rank sum test was used to determine differences in ordered variables (e.g., stage) in the distribution of the two groups. Continuous variables were transformed into categorical variables for processing. Using univariate and multivariate Cox regression analyses, the independent predictors for LRFS and OS were identified. These statistically significant factors (P<0.05) identified by univariate analysis will be incorporated into the multivariate analysis. Subsequently, nomograms were created to predict LRFS and OS based on the independent predictors identified by multivariate Cox regression analyses. Additionally, the bootstrap method was used for internal and external validation to evaluate the performance of the prediction model. The bootstrap-corrected OS rates were calculated by averaging the Kaplan−Meier estimates based on 2,000 bootstrap samples. The discrimination and calibration of the nomogram were evaluated using calibration curves. Receiver operating characteristic (ROC) curves and calibration curves were constructed to assess the precision of the nomogram. R software version 4.0.1 was used for the Cox proportional hazard regression analysis and construction and evaluation of the nomogram. Two-sided statistical analysis was used to calculate all P values.

**Table 1:** Baseline clinical and pathological characteristics of all the enrolled patients.

Characteristic	Overall cohort N=903	Testing set N=301	Training set N=602	p
Sex, No. (%)				
Female	351 (38.87)	134 (44.52)	275 (45.68)	0.795
Male	552 (61.13)	167 (55.48)	327 (54.32)	
Age at diagnosis, No. (%), years				
<65	409 (45.29)	111 (36.88)	240 (39.87)	0.426
>=65	494 (54.71)	190 (63.12)	362 (60.13)	
Tumor location, No. (%)				
Colon tumor	527 (58.36)	176 (58.47)	353 (58.64)	0.632
Rectum tumor	376 (41.64)	125 (41.53)	249 (41.36)	
Tumor pathological type, No. (%)				
Adenocarcinoma	713 (78.96)	244 (81.06)	469 (77.91)	0.312
Mucinous/Signet	190 (21.04)	57 (18.94)	133 (22.09)	
Tumor size, No. (%), cm				
<=3	118 (13.07)	36 (11.96)	82 (13.62)	0.688
(3-5)	401 (44.41)	132 (43.85)	269 (44.68)	
>=5	384 (42.52)	133 (44.19)	251 (41.69)	
Vascular invasion, No. (%)				
No	611 (78.64)	202 (78.29)	409 (78.81)	0.944
Yes	166 (21.36)	56 (21.71)	110 (21.19)	
Perineural invasion, No. (%)				
No	505 (67.97)	160 (66.39)	345 (68.73)	0.579
Yes	238 (32.03)	81 (33.61)	157 (31.27)	
CEA, No. (%)				
<5 ng/mL	606 (67.11)	194 (64.45)	412 (68.44)	0.26
>=5 ng/mL	297 (32.89)	107 (35.55)	190 (31.56)	
CA199, No. (%)				
<37 IU/mL	612 (67.77)	202 (67.11)	410 (68.11)	0.821
>=37 IU/mL	291 (32.23)	99 (32.89)	192 (31.89)	
Tumor differentiation grade, No. (%)				
Grade(1/2)	682 (75.53)	225 (74.75)	457 (75.91)	0.763
Grade(3/4)	221 (24.47)	76 (25.25)	145 (24.09)	
Pathological T stage, No. (%)				
T1/2	144 (15.95)	47 (15.61)	97 (16.11)	0.327
T3/4	759 (84.05)	254 (84.39)	505 (83.89)	
Pathological N stage, No. (%)				
N0	689 (76.30)	226 (75.08)	463 (76.91)	0.599
N1/2	214 (23.70)	75 (24.92)	139 (23.09)	
No. of harvested lymph nodes, No. (%)				
>=12	830 (91.92)	274 (91.03)	556 (92.36)	0.575
<12	73 (8.08)	27 (8.97)	46 (7.64)	
Adjuvant therapy, No. (%)				
No	350 (38.76)	125 (41.53)	225 (37.38)	0.02
Before surgery	90 (9.97)	39 (12.96)	51 (8.47)	
After surgery	463 (51.27)	137 (45.51)	326 (54.15)	

CEA, carcinoembryonic antigen; CA199, carbohydrate antigen 199.

## 3 Results

### 3.1 Patient characteristics

A total of 903 stage II-III CRC patients [351 (38.87%) women and 552 (61.13%) men; mean age, 60 years ± 11 (SD)] who underwent radical surgery were enrolled from the CNCC set. Among these patients, 136 suffered from locally recurrent CRC within 100 months after the first surgery. The main histological type of tumor was adenocarcinoma (78.96%). For tumor size, many patients (44.41%) had tumor sizes ranging from 3 cm to 5 cm, followed by 5-10 cm (42.52%). With regard to tumor differentiation grade, most patients (75.53%) had tumors in grade 1 or 2. The patients from the CNCC were divided into training and testing sets, as shown in **Table 1**.

### 3.2 Predictive factors for recurrence and survival

The Cox regression analysis was applied in the CNCC set to identify the predictors of LRFS and OS. Univariate analysis indicated that vascular invasion [hazard ratio (HR): 2.99; 95% confidence interval (CI): 1.99-4.48, p<0.001], perineural invasion (2.22, 1.49-3.33, p<0.001), tumor grade (1.85, 1.30-2.63, p<0.001), T stage (3.81, 2.34-6.20, p<0.001), N stage (3.36, 2.40-4.72, p<0.001), and harvested lymph nodes (1.74, 1.05-2.87, p=0.031) were correlated with LRFS in the patients with CRC (all p<0.05) (**Table 2**). Furthermore, the multivariate Cox analysis demonstrated that vascular invasion (1.67, 1.04-2.68, p=0.033), perineural invasion (1.65, 1.06-2.58, p=0.027), T stage (5.72, 2.86-11.41, p<0.001), N stage (2.43, 1.51-3.89, p<0.001), and harvested lymph nodes (2.78, 1.50-5.13, p=0.001) were independent risk factors for LRFS. Thus, these variables were included in the predictive model (**Table 2**). Age at diagnosis (2.59, 1.81-3.69, p<0.001), tumor pathological type (1.50, 1.07-2.12, p=0.019), vascular invasion (2.58, 1.71-3.88, p<0.001), perineural invasion (2.54, 1.70-3.79, p<0.001), tumor grade (1.51, 1.08-2.12, p=0.017), T stage (2.94, 1.94-4.45, p<0.001), N stage (3.83, 2.79-5.26, p<0.0001) and harvested lymph nodes (2.45, 1.61-3.74, p<0.001) were correlated with OS in the patients with CRC (all p < 0.05) (**Table 2**). In addition, the multivariate Cox analysis demonstrated that age at diagnosis (2.58, 1.63-4.09, p<0.001), tumor pathological type (1.67, 1.01-2.77, p=0.047), vascular invasion (1.99, 1.23-3.22, p=0.005), perineural invasion (1.64, 1.05-2.57, p=0.030), T stage (6.09, 3.04-12.19, p<0.001), N stage (2.48, 1.55-3.96, p<0.001), and harvested lymph nodes (2.91, 1.56-5.44, p=0.001) were independent risk factors for OS. Thus, these variables were included in the predictive model (**Table 2**).

**Table 2:** Univariable and multivariable regression analyses of risk factors for local recurrence-free survival and overall survival in all 903 patients.

Characteristic	Overall survival				Local recurrence-free survival			
	Univariable analysis		Multivariate analysis		Univariable analysis		Multivariate analysis	
	HR (95%CI)	*P*	HR (95%CI)	*P*	HR (95%CI)	*P*	HR (95%CI)	*P*
Sex								
Female	Ref							
Male	1.13 (0.82-1.56)	0.465			1.43 (1.00-2.05)	0.051		
Age at diagnosis, years								
<60	Ref							
>=60	2.59 (1.81-3.69)	<0.001	2.58 (1.63-4.09)	<0.001	1.15 (0.82-1.62)	0.411		
Tumor location								
Colon tumor	Ref							
Rectum tumor	1.38 (0.97-1.97,	0.075			0.92 (0.64-1.31)	0.63		
Tumor pathological type,								
Adenocarcinoma	Ref							
Mucinous adenocarcinoma	1.50 (1.07-2.12)	0.019	1.67 (1.01-2.77)	0.047	1.11 (0.75-1.64)	0.614		
Tumor size, cm								
<=3	Ref							
(3-5)	0.70 (0.45-1.08)	0.108			0.59 (0.37-1.05)	0.069		
>=5	0.60 (0.39-1.04)	0.076			0.63 (0.40-1.01)	0.055		
Vascular invasion,								
No	Ref							
Yes	2.58 (1.71-3.88)	<0.001	1.99 (1.23-3.22)	0.005	2.99 (1.99-4.48)	<0.001	1.67 (1.04-2.68)	0.033
Perineural invasion								
No	Ref							
Yes	2.54 (1.70-3.79)	0.001	1.64 (1.05-2.57)	0.030	2.22 (1.49-3.33)	<0.001	1.65 (1.06-2.58)	0.027
CEA								
Negative	Ref							
Positive	1.22 (0.88-1.67)	0.230			1.14 (0.81-1.62)	0.447		
CA199								
Negative	Ref							
Positive	1.06 (0.77-1.47)	0.726			1.14 (0.80-1.61)	0.466		
Tumor grade								
Grade (1/2)	Ref							
Grade (3/4)	1.51 (1.08-2.12)	0.017	1.12 (0.71-1.77)	0.626	1.85 (1.30-2.63)	<0.001	1.32 (0.85-2.05)	0.211
Tumor pathological T stage								
T1/2_stage	Ref							
T3/4_stage	2.94 (1.94-4.45)	<0.001	6.09 (3.04-12.19)	<0.001	3.81 (2.34-6.20)	<0.001	5.72 (2.86-11.41)	<0.001
Tumor pathological N stage								
N0_stage	Ref							
N1/2_stage	3.83 (2.79-5.26)	<0.001	2.48 (1.55-3.96)	<0.001	3.36 (2.40-4.72)	<0.001	2.43 (1.51-3.89)	<0.001
No. of harvested lymph nodes, No.								
>=12	Ref							
<12	2.45 (1.61-3.74)	<0.001	2.91 (1.56-5.44)	0.001	1.74 (1.05-2.87)	0.031	2.78 (1.50-5.13)	0.001
Adjuvant therapy								
No	Ref							
Before surgery	1.38 (0.86-2.22)	0.186			0.97 (0.53-1.76)	0.91		
After surgery	0.78 (0.56-1.09)	0.142			1.00 (0.70-1.42)	0.982		

CEA, carcinoembryonic antigen; CA199, carbohydrate antigen 199.

### 3.3 The prognostic impact of adjuvant therapy on patients with CRC

**Figure 2:**
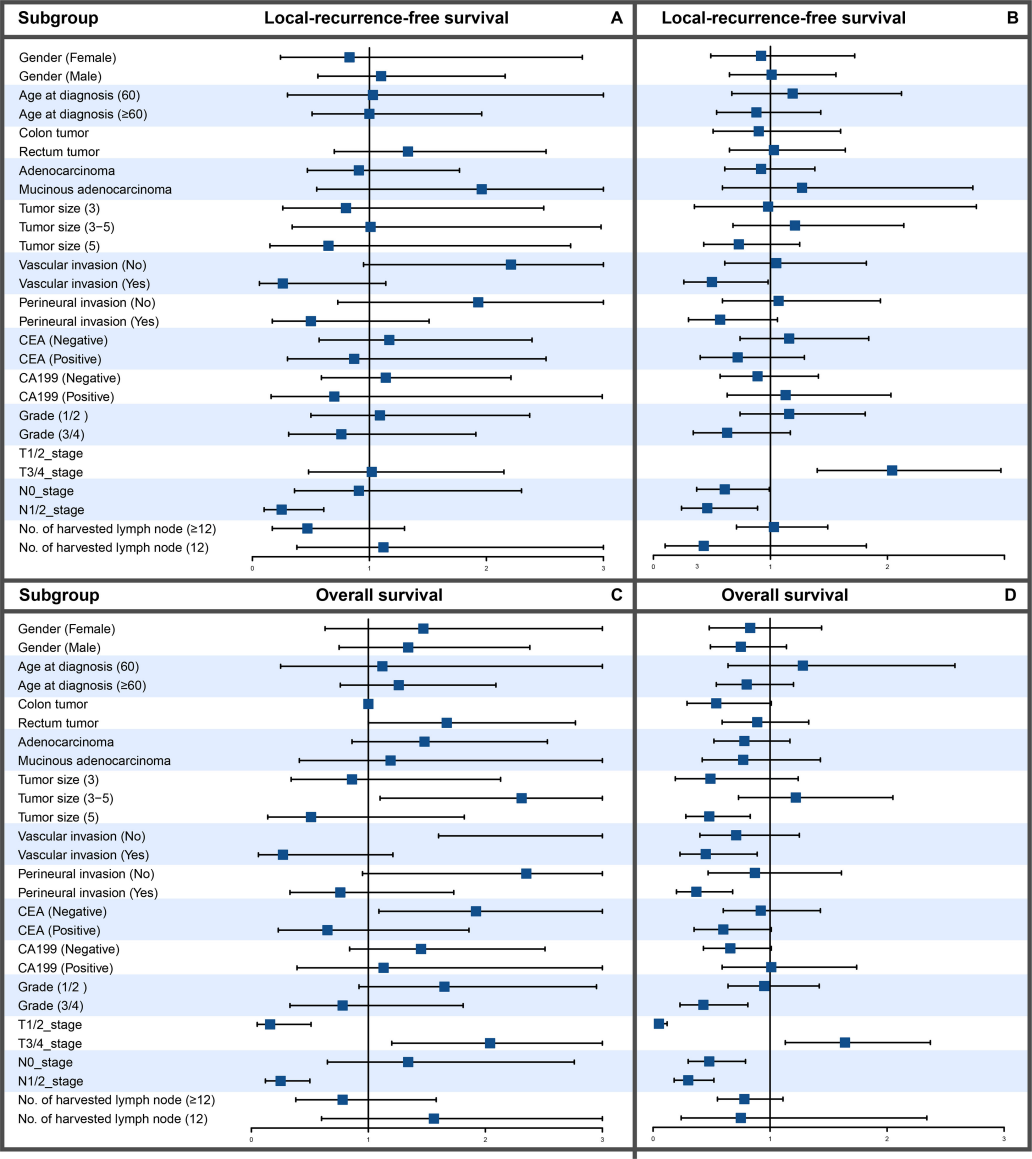
Subgroup analysis of LRFS and OS between patients with CRC treated with and without chemotherapy. **(A)** LRFS in chemotherapy patients. **(B)** LRFS in patients without chemotherapy. **(C)** OS in chemotherapy patients. **(D)** OS in patients without chemotherapy.

To evaluate the efficacy of adjuvant therapy for influencing recurrence and survival, we analyzed the prognosis before and after surgery for LRFS and OS. The Cox regression analysis suggested that adjuvant therapy did not influence the prognosis (**Table 2**). The subgroup analysis showed that adjuvant therapy before surgery improved the LRFS and OS for patients with N1- or 2-stage CRC and adjuvant therapy after surgery improved the OS for patients with vascular invasion, perineural invasion, or grade 3 or 4 (**Figures 2A–D**). Baseline characteristics comparisons of patients with CRC between the adjuvant and no adjuvant therapy groups were performed before and after propensity score matching (PSM) using the CNCC set (**Supplementary Tables S1**, **S2**). To compare the effectiveness between the no adjuvant therapy and adjuvant therapy before surgery groups, we identified matching patients with CRC after PSM. Notably, there was no significant difference in LRFS and OS between the no adjuvant and adjuvant therapy before surgery groups, both before and after PSM, with all P values >0.05 (**Supplementary Figure S1**). Similarly, there was no significant difference in LRFS and OS between the no adjuvant and adjuvant therapy groups after surgery, with all P values >0.05 (**Supplementary Figure S2**).

### 3.4 Nomogram construction and validation

**Figure 3:**
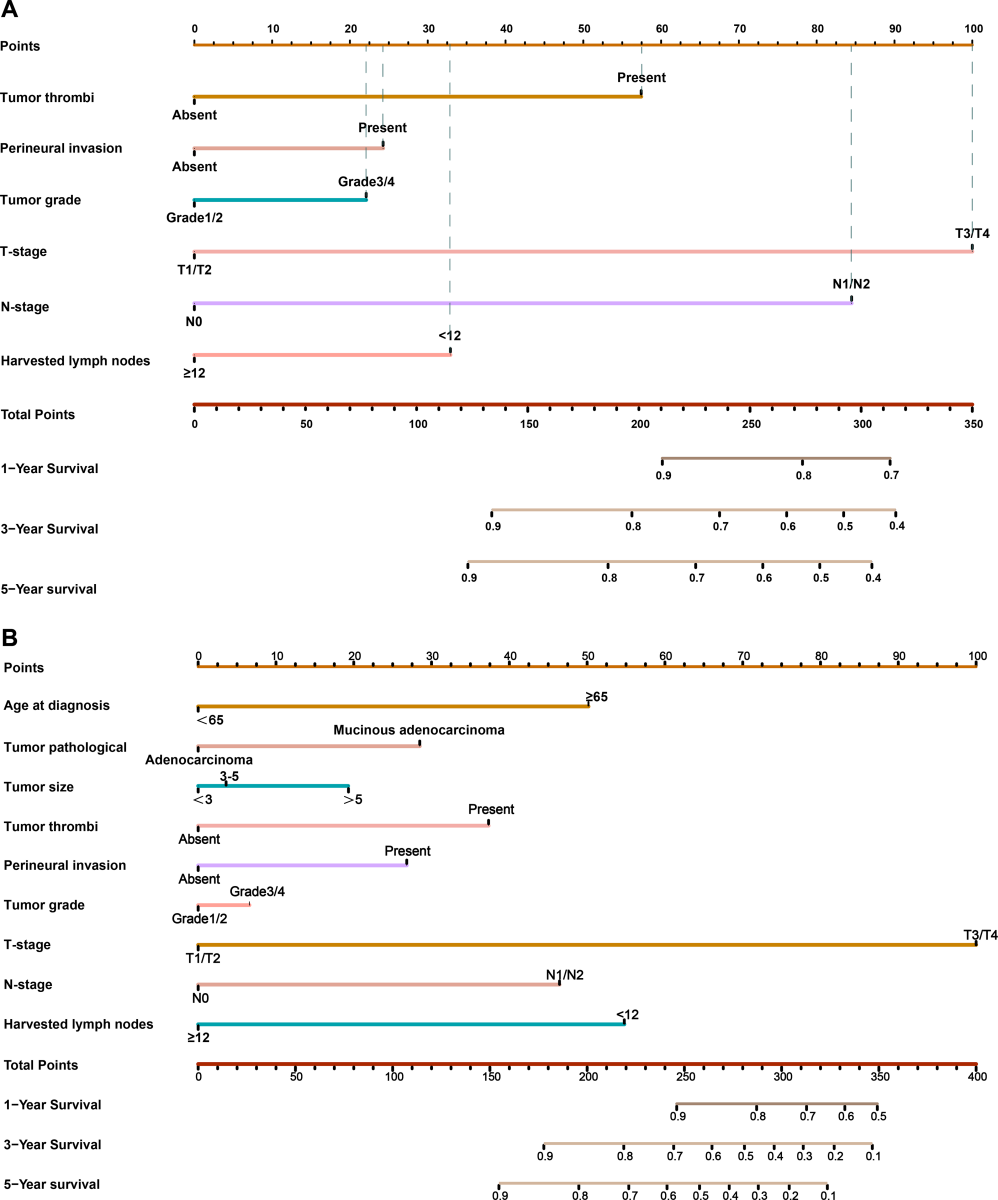
Nomogram for predicting 1-, 3- and 5-year LRFS and OS after surgery. **(A)** Nomogram for LRFS in all patients. **(B)** Nomogram for OS in all patients. Online tool is available at https://oldcoloncancer.shinyapps.io/dynnomapp/.

Our results showed that adjuvant therapy could not predict the LRFS and OS for CRC patients in the CNCC set. We constructed nomogram models to predict LRFS and OS between the two sets (training sets and test sets). Vascular invasion, perineural invasion, T stage, N stage, and number of harvested lymph nodes were identified as independent risk factors for LRFS by the multivariate analysis. The risk factors that were correlated with LRFS were incorporated into the nomogram development (**Figure 3A**). In addition, age at diagnosis, tumor pathological type, vascular invasion, perineural invasion, T stage, N stage, and number of harvested lymph nodes were identified as independent risk factors for OS by the multivariate analysis. These risk factors correlated with OS were incorporated into the development of the nomogram (**Figure 3B**, **Supplementary Figure S3**). The prognostic nomogram for patients with CRC was created in the training set, which was then validated in the testing set. To add clinical convenience, a user-friendly online application for the two nomograms was developed and uploaded to our website (https://oldcoloncancer.shinyapps.io/dynnomapp/).

### 3.5 Survival risk stratification

To facilitate the use in clinical practice of these nomogram models, we divided the patients with CRC into two risk groups according to the risk scores of the nomogram models: a high-risk group and a low-risk group. We identified the cutoff values using the median risk scores in the training set and verified them in the test set. This pragmatic visualization of the risk level could help decide the optimal treatment strategy for patients with CRC. According to the risk scores from both nomogram models, the LRFS and OS of the patients in the low-risk group were better than those of the patients in the high-risk group in all sets (log-rank test, *P* < 0.05). Subgroup analysis was performed to evaluate the performance of these risk groups in different subgroups. The LRFS and OS of the patients in the low-risk group were better than those in the high-risk group (**Supplementary Figure S4**).

### 3.6 Comparison of the predictive accuracy of nomograms

**Figure 4:**
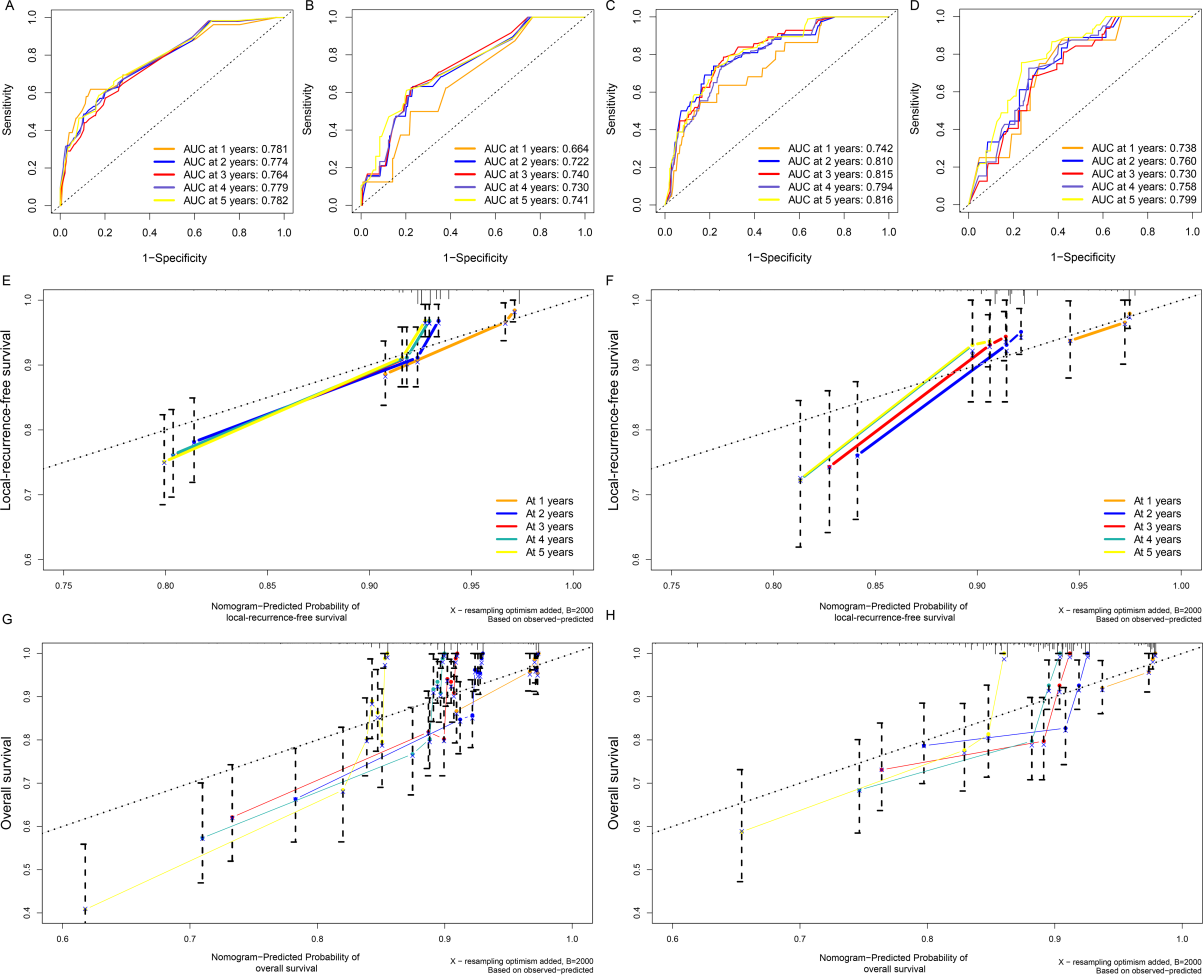
The AUCs of the nomogram model in **(A)** the training cohort and **(B)** the testing cohort for predicting LRFS and in **(C)** the training cohort **(D)** and testing cohort for predicting OS. The nomogram model calibration curves in **(E)** the training cohort **(F)** and the testing cohort for predicting LRFS and in **(G)** the training cohort and **(H)** the testing cohort for predicting OS.

To evaluate the discrimination ability of the two nomograms, we evaluated the AUCs (**Figures 4A–D**). We evaluated the relationship between the OS rates and the predicted probabilities in all models and staging systems. The bootstrapped calibration curves plotted with 1-,2-, 3-,4- and 5-year LRFS and OS were well matched with the idealized 45° line for both the nomograms (**Figures 4E–H**).

### 3.7 Risk stratification for patients with CRC

**Figure 5:**
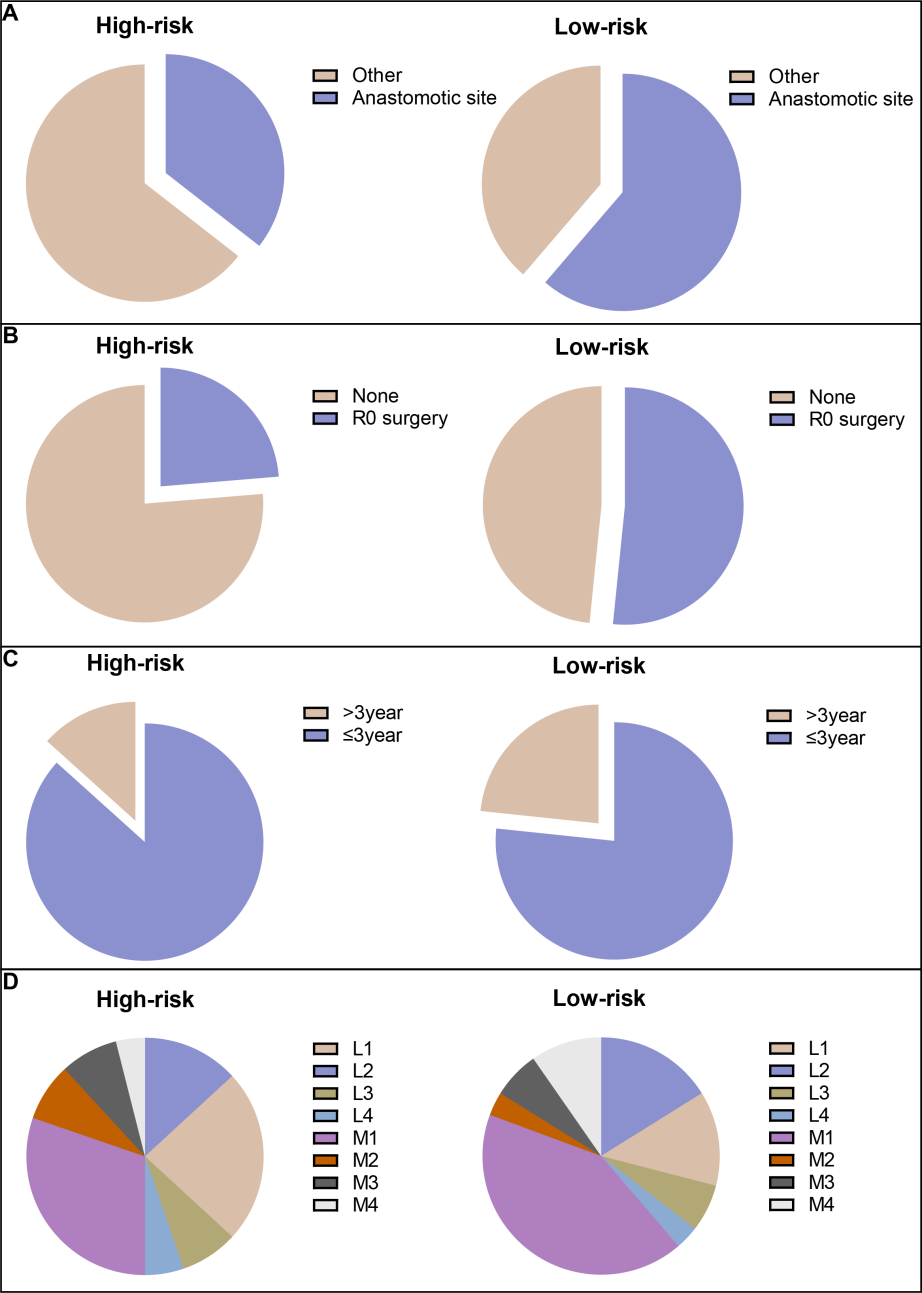
The risk stratification for CRC patients. **(A)** Locally recurrent colon cancer categorized as peri-anastomotic; **(B)** patients with locally recurrent colon cancer received R0 surgery; **(C)** 3-year LRFS; and **(D)** recurrent sites.

Among the patients in all risk groups, 250 (51.87%) in the high-risk group and 244 (57.96%) in the low-risk group were older than 60 years (Chi-squared test; p=0.077) according to the model for LRFS. There were more patients with locally recurrent colon cancer categorized as peri-anastomotic in the low-risk group than in the high-risk group (**Figure 5A**). A higher percentage of patients with locally recurrent colon cancer received R0 surgery in the low-risk group than in the high-risk group (**Figure 5B**). In addition, there were no significant differences in 3-year LRFS or recurrence sites between the two groups (**Figures 5C, D**).

## 4 Discussion

Despite significant advances in the management of TME surgery procedures coupled with standard adjuvant chemotherapy, a considerable number of patients with CRC still have local recurrence, which seriously affects their long-term survival (17). Surgical intervention to treat locally recurrent CRC has been steadily gaining momentum, but it remains highly morbid and challenging to manage. Unlike distant metastases (distant recurrence), in which secondary malignant tumors grow beyond the initial organ in solid organs such as the liver, lungs, and brain, locally recurrent diseases are confined to the original surgery site and regional lymph nodes. However, locally recurrent diseases can cause severe symptoms, such as intestinal obstruction and perforation, that require timely treatment. Individualized prediction and treatment suggestions for postoperative recurrence risk in patients with CRC are necessary, which may improve OS in high-risk patients. Currently, predicting the recurrence risk and survival of patients with CRC is typically done using the TNM staging system, but its precision has limitations.

We identified six variables—tumor thrombi, perineural invasion, tumor grade, T stage, N stage, and harvested lymph nodes—as critical determinants of local recurrence outcomes to model the clinical risk calculator (**Supplementary Figure S3**). In our cohort, CRC patients with tumor thrombi, perineural invasion, and tumor grade 1 or 2 had relatively poor LRFS and OS, which agrees with previous reports (18, 19). pT stage and pN stage account for a large weight in our nomogram model, but the overall predictive ability of the model was better than that of the TNM staging system alone. Only 10% of patients with pT1 have lymph node metastasis (20). Our study shows that patients with pT3/4 stage had a higher risk of local recurrence than pT1/2 patients, and there were significant differences in both the univariate and multivariate analyses (p<0.01). In addition, previous studies have shown that some clinicopathological factors, such as gender, age, neoadjuvant rectal score, and even tumor grade, may also influence the prognosis of patients (21, 22). However, this study did not find this significant difference. Mucinous adenocarcinoma comprises approximately 5% to 20% of all CRC cases but the prognosis remains controversial (23, 24). Some studies have reported that patients with mucinous adenocarcinoma have a poorer survival rate than patients with non-mucinous adenocarcinoma, whereas others have reported survival similar to those of patients with non-mucinous adenocarcinoma (25). In this study, approximately 20% of the patients had mucinous adenocarcinoma, which had poor overall survival and similar local recurrence-free survival compared with the patients with non-mucinous adenocarcinoma.

When considering preoperative or postoperative adjuvant therapy, no significant improvement due to perioperative adjuvant therapies of LRFS and OS in patients with resectable stage II-III CRC was observed in the present dataset. However, subgroup analysis showed that preoperative adjuvant therapy can improve LRFS and OS in T3/4 patients, as shown in **Figure 2**. Postoperative adjuvant therapy was administered in more than half (51.8%) of the patients, and preoperative adjuvant therapy was administered in less than one-tenth (9.98%). To account for possible differences in baseline data, such as the location and stage of the tumor, we further conducted a PSM analysis (**Supplementary Table S1**). The results were consistent with those before PSM. Perioperative adjuvant therapy did not significantly improve LRFS or OS for patients with stage II-III CRC (**Supplementary Figure S2**). The possible reason is that our study classified adjuvant chemotherapy and radiotherapy as adjuvant therapy. (Neo)adjuvant radiotherapy is usually performed in patients with locally advanced rectal cancer, which may impact the prognosis of rectal cancer (18). However, patients who receive adjuvant therapy are generally stage III and high-risk stage II. In contrast, recurrent and metastatic diseases that develop after adjuvant therapy could resist radiotherapy and chemotherapy, resulting in a poor prognosis after the disease appears (26). The benefit of adjuvant chemotherapy on overall survival in stage III CRC has been well established, but whether adjuvant chemotherapy is needed for patients with stage II CRC is still controversial (27–29). Hence, our model did not incorporate the factor of adjuvant therapy.

Over the past few years, numerous prediction models, such as nomograms, have been created to predict the prognosis of CRC, possibly owing to their high utility in daily clinical practice. The reported nomogram AUC values for stages I to III CRC ranged from 0.68 to 0.80 (12, 30). Our Prediction Tools web interface provides the most efficient method for using the calculator by incorporating estimates from the Kaplan−Meier curves for patients from the nomogram for all other patients, showing better predictive power and discrimination. The AUC value of the LRFS nomogram was 0.764 in the training cohort and 0.740 in the validation cohort and that of the OS nomogram was 0.815 in the training cohort and 0.730 in the validation cohort, indicating good predictability compared with preexisting nomograms. In further analysis, we divided all patients into high-risk and low-risk groups according to the established model for subgroup analysis, and the results showed that there were significant differences in each subgroup (**Supplementary Figure S4**), proving that our model has good predictive performance. We conducted a novel analysis in which stratification was carried out according to the risk of recurrence in patients to predict the possible treatment decisions for patients after recurrence. We concluded that low-risk patients were more likely to undergo salvage radical surgery (R0). In the future, we will conduct further verification in a larger clinical cohort to achieve better clinical application to guide the individualized treatment of patients.

Notably, the clinical risk calculator utilized in this study was developed using data from a single institution in China, which may have resulted in some degree of selection bias due to the retrospective nature of the study. In recent years, there has been a growing focus on precision medicine that utilizes biomarkers such as microsatellite instability status, Ras mutations, and consensus molecular subtypes (17). However, our database lacks this information. Adherence to standards is particularly notable given that a recent study discovered the majority of prognostic tools for CRC are methodologically deficient (31). Furthermore, our study covers a relatively long period, and different periods may have differences between the pathological diagnosis and post-recurrence treatment. Although there was no difference in the baseline characteristics of the patients in each group, this long period may still affect the statistical analysis results. In addition, it should be noted that approximately 10% of the study population were patients who received neoadjuvant therapy, which may downstage the pathology of these patients compared to their pretreatment stage. However, these patients had a negligible impact on the results because there were no statistical differences in univariate and multivariate Cox analysis. Future external validation will help refine the calibration.

## 5 Conclusion

The clinical risk calculator can better account for tumor and patient heterogeneity, providing a more individualized outcome prognostication for LRFS and OS. By identifying high- and low-risk patients, the model is expected to aid in treatment planning, such as resectability evaluation; it can also be used in postoperative surveillance (https://oldcoloncancer.shinyapps.io/dynnomapp/).

## Data Availability

The original contributions presented in the study are included in the article/**Supplementary Material**. Further inquiries can be directed to the corresponding authors.
