# Supplementary figures and images for: Clinical calculator based on clinicopathological characteristics predicts local recurrence and overall survival following radical resection of stage II-III colorectal cancer
Source: Front Oncol. 2025 Feb 5;15:1494255. doi: 10.3389/fonc.2025.1494255 (PMC11835698; doi:10.3389/fonc.2025.1494255)

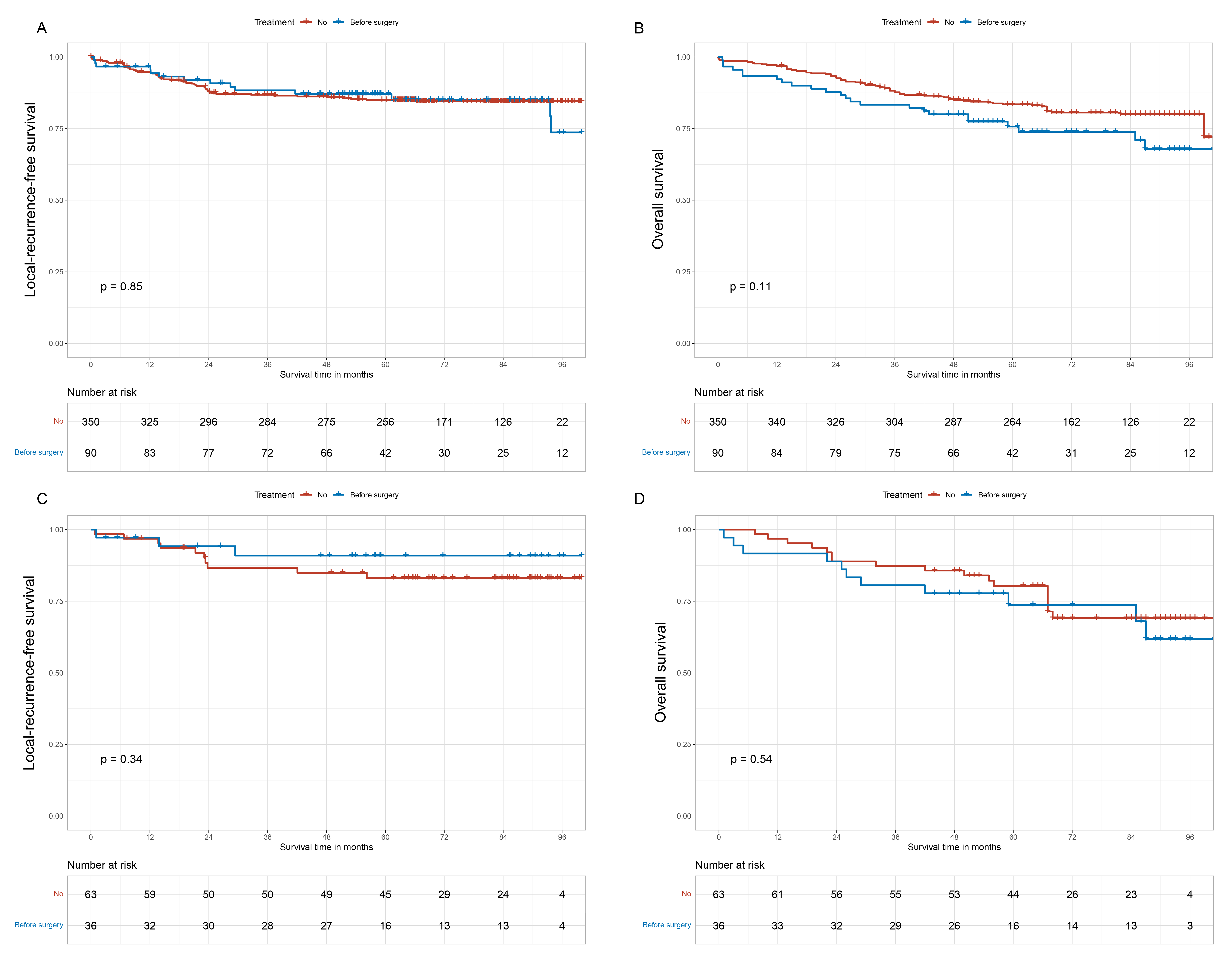

Supplement: Supplementary file 1 [file Image1.tif]

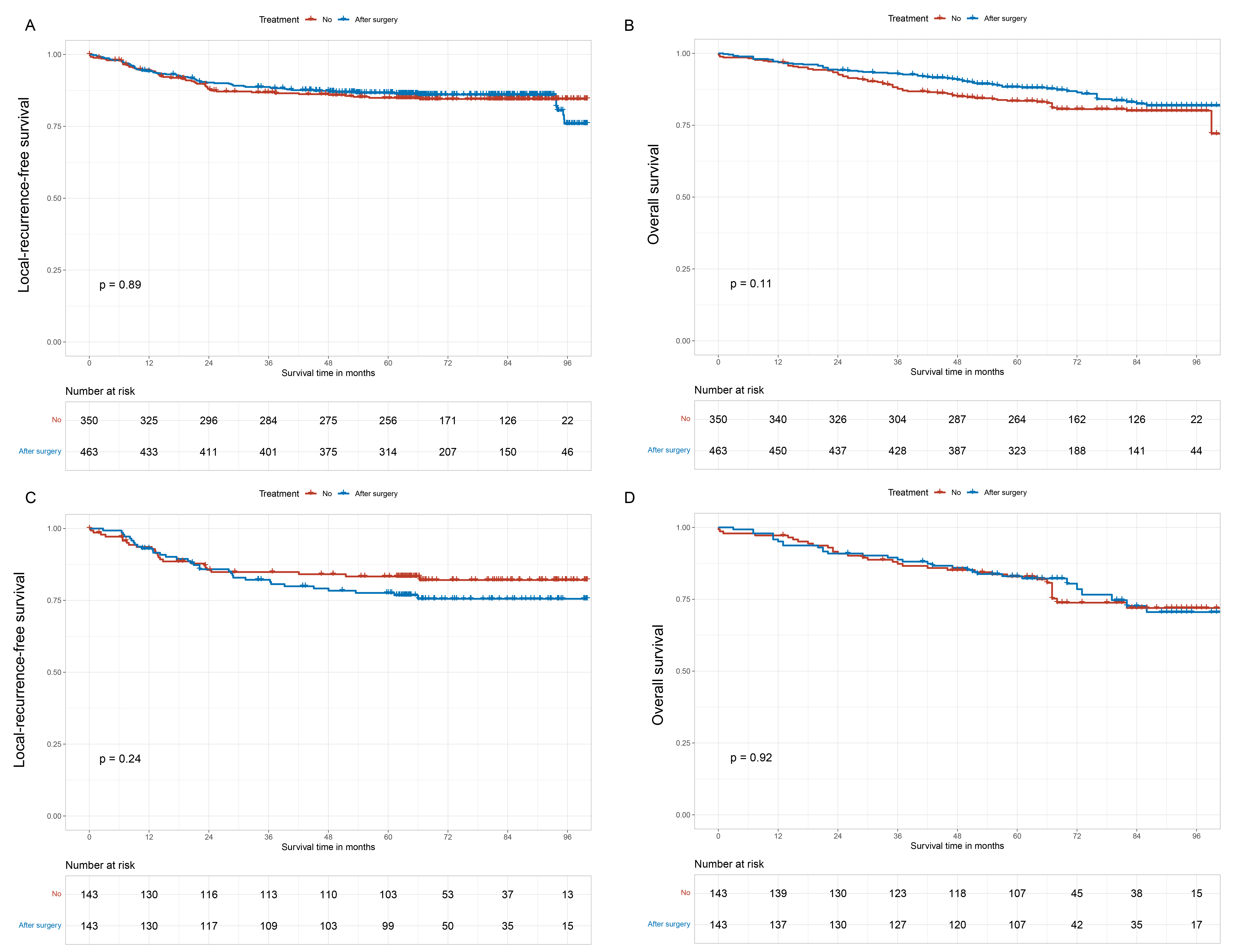

Supplement: Supplementary file 2 [file Image2.tif]

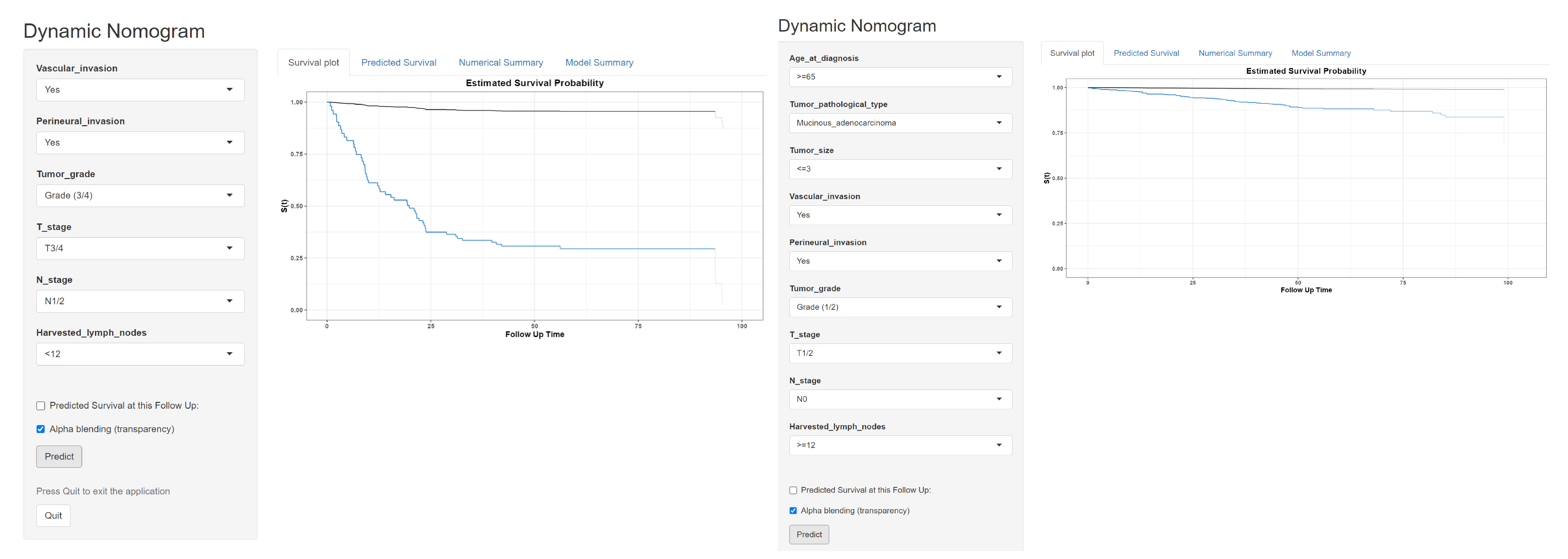

Supplement: Supplementary file 3 [file Image3.tif]

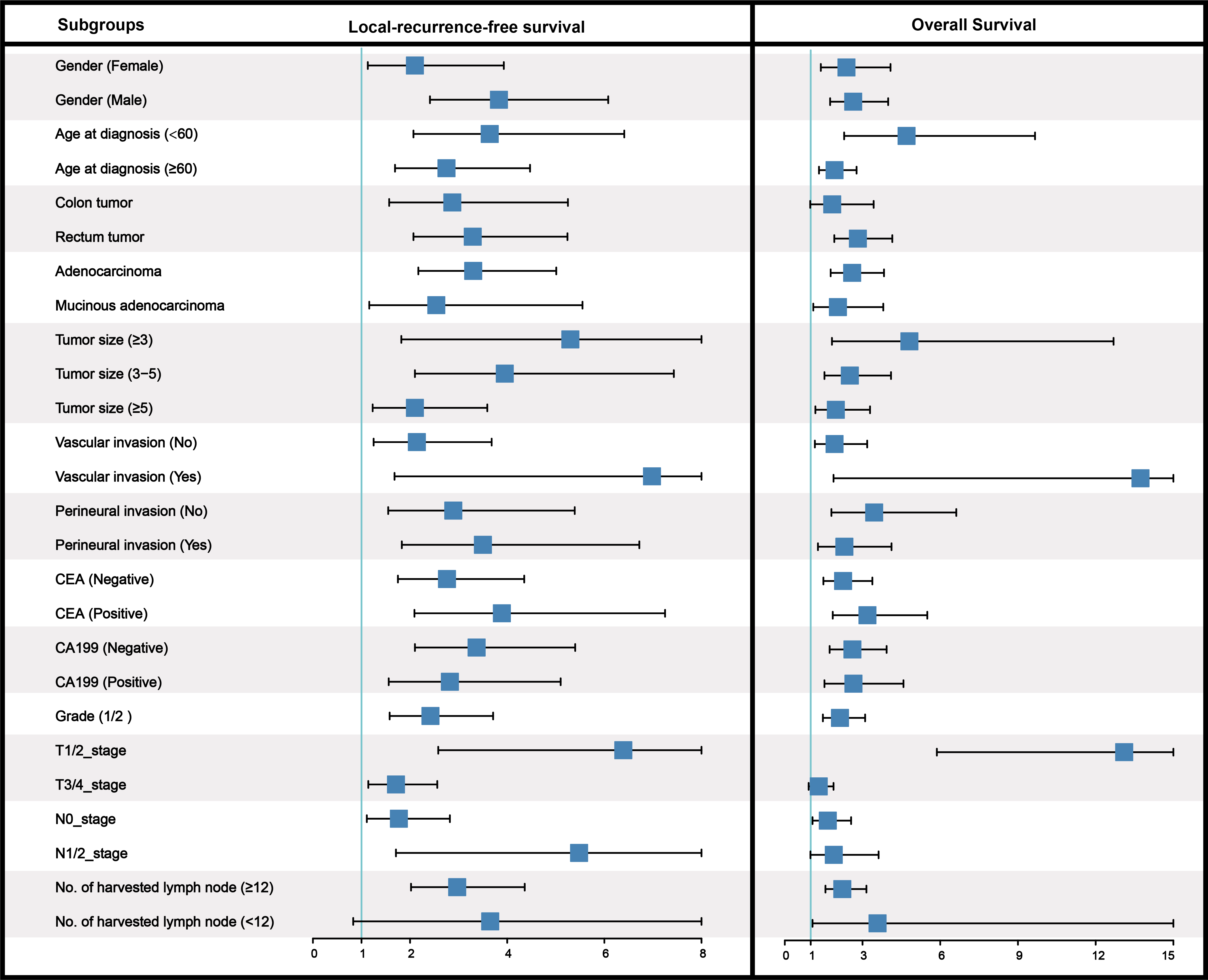

Supplement: Supplementary file 4 [file Image4.tif]
