# Supplementary material for: Clinical calculator based on clinicopathological characteristics predicts local recurrence and overall survival following radical resection of stage II-III colorectal cancer
Source: Front Oncol. 2025 Feb 5;15:1494255. doi: 10.3389/fonc.2025.1494255 (PMC11835698; doi:10.3389/fonc.2025.1494255)
Supplement: Supplementary file 5 [file Table1.docx]

**Supplement Table 1. Comparison of baseline characteristics between preoperative adjuvant therapy and no therapy groups before and after PSM**

| Characteristics | Before PSM | | p | After PSM | | p |
| --- | --- | --- | --- | --- | --- | --- |
|  | No therapy N=350 | Adjuvant therapy N=90 |  | No therapy  N=63 | Adjuvant therapy N=36 |  |
| Gender, No. (%) |  |  |  |  |  |  |
| Female | 133 (38.00) | 29 (32.22) | 0.373 | 22 (34.92) | 16 (44.44) | 0.47 |
| Male | 217 (62.00) | 61 (67.78) |  | 41 (65.08) | 20 (55.56) |  |
| Age at diagnosis, No. (%), years |  |  |  |  |  |  |
| <65 | 128 (36.57) | 22 (24.44) | 0.041 | 25 (39.68) | 15 (41.67) | 1 |
| >=65 | 222 (63.43) | 68 (75.56) |  | 38 (60.32) | 21 (58.33) |  |
| Tumor location, No. (%) |  |  |  |  |  |  |
| Colon tumor | 126 (36.00) | 12 (13.33) | <0.001 | 19 (30.16) | 10 (27.78) | 0.983 |
| Rectum tumor | 224 (64.00) | 78 (86.67) |  | 44 (69.84) | 26 (72.22) |  |
| Tumor pathological type, No. (%) |  |  |  |  |  |  |
| Adenocarcinoma | 269 (76.86) | 76 (84.44) | 0.157 | 54 (85.71) | 31 (86.11) | 1 |
| Mucinous adenocarcinoma | 81 (23.14) | 14 (15.56) |  | 9 (14.29) | 5 (13.89) |  |
| Tumor size, No. (%), cm |  |  |  |  |  |  |
| <=3 | 23 (6.57) | 37 (41.11) | <0.001 | 7 (11.11) | 7 (19.44) | 0.519 |
| (3-5) | 159 (45.43) | 36 (40.00) |  | 35 (55.56) | 18 (50.00) |  |
| >=5 | 168 (48.00) | 17 (18.89) |  | 21 (33.33) | 11 (30.56) |  |
| Vascular invasion, No. (%) |  |  |  |  |  |  |
| No | 289 (89.20) | 47 (74.60) | 0.003 | 46 (85.19) | 18 (64.29) | 0.059 |
| Yes | 35 (10.80) | 16 (25.40) |  | 8 (14.81) | 10 (35.71) |  |
| Perineural invasion, No. (%) |  |  |  |  |  |  |
| No | 248 (81.31) | 32 (51.61) | <0.001 | 41 (78.85) | 15 (55.56) | 0.057 |
| Yes | 57 (18.69) | 30 (48.39) |  | 11 (21.15) | 12 (44.44) |  |
| CEA, No. (%) |  |  |  |  |  |  |
| Negative | 235 (67.14) | 66 (73.33) | 0.318 | 44 (69.84) | 28 (77.78) | 0.536 |
| Positive | 115 (32.86) | 24 (26.67) |  | 19 (30.16) | 8 (22.22) |  |
| CA199, No. (%) |  |  |  |  |  |  |
| Negative | 223 (63.71) | 73 (81.11) | 0.003 | 48 (76.19) | 28 (77.78) | 1 |
| Positive | 127 (36.29) | 17 (18.89) |  | 15 (23.81) | 8 (22.22) |  |
| Tumor grade, No. (%) |  |  |  |  |  |  |
| Grade (1/2 ) | 274 (78.29) | 62 (68.89) | 0.083 | 43 (68.25) | 25 (69.44) | 1 |
| Grade (3/4) | 76 (21.71) | 28 (31.11) |  | 20 (31.75) | 11 (30.56) |  |
| Tumor pathological T stage, No. (%) |  |  |  |  |  |  |
| T1/2_stage | 6 (1.71) | 35 (38.89) | <0.001 | 6 (9.52) | 3 (8.33) | 1 |
| T3/4_stage | 344 (98.29) | 55 (61.11) |  | 57 (90.48) | 33 (91.67) |  |
| Tumor pathological N stage, No. (%) |  |  |  |  |  |  |
| N0_stage | 326 (93.14) | 42 (46.67) | <0.001 | 48 (76.19) | 24 (66.67) | 0.43 |
| N1/2_stage | 24 (6.86) | 48 (53.33) |  | 15 (23.81) | 12 (33.33) |  |
| No. of harvested lymph nodes, No. (%) |  |  |  |  |  |  |
| >=12 | 332 (94.86) | 58 (64.44) | <0.001 | 54 (85.71) | 31 (86.11) | 1 |
| <12 | 18 (5.14) | 32 (35.56) |  | 9 (14.29) | 5 (13.89) |  |

Abbreviation: CEA, carcinoembryonic antigen; CA199, Carbohydrate antigen 199.
