# Supplementary material for: Clinical calculator based on clinicopathological characteristics predicts local recurrence and overall survival following radical resection of stage II-III colorectal cancer
Source: Front Oncol. 2025 Feb 5;15:1494255. doi: 10.3389/fonc.2025.1494255 (PMC11835698; doi:10.3389/fonc.2025.1494255)
Supplement: Supplementary file 6 [file Table2.docx]

**Supplement Table 2. Comparison of baseline characteristics between postoperative adjuvant therapy and no therapy groups before and after PSM**

| Characteristics | Before PSM | | p | After PSM | | p |
| --- | --- | --- | --- | --- | --- | --- |
|  | No therapy N=350 | Adjuvant therapy N=463 |  | No therapy N=143 | Adjuvant therapy N=143 |  |
| Gender, No. (%) |  |  |  |  |  |  |
| Female | 133 (38.00) | 189 (40.82) | 0.458 | 58 (40.56) | 50 (34.97) | 0.393 |
| Male | 217 (62.00) | 274 (59.18) |  | 85 (59.44) | 93 (65.03) |  |
| Age at diagnosis, No. (%), years |  |  |  |  |  |  |
| <65 | 128 (36.57) | 259 (55.94) | <0.001 | 81 (56.64) | 87 (60.84) | 0.548 |
| >=65 | 222 (63.43) | 204 (44.06) |  | 62 (43.36) | 56 (39.16) |  |
| Tumor location, No. (%) |  |  |  |  |  |  |
| Colon tumor | 126 (36.00) | 151 (32.61) | 0.35 | 55 (38.46) | 54 (37.76) | 1 |
| Rectum tumor | 224 (64.00) | 312 (67.39) |  | 88 (61.54) | 89 (62.24) |  |
| Tumor pathological type, No. (%) |  |  |  |  |  |  |
| Adenocarcinoma | 269 (76.86) | 368 (79.48) | 0.416 | 106 (74.13) | 102 (71.33) | 0.69 |
| Mucinous adenocarcinoma | 81 (23.14) | 95 (20.52) |  | 37 (25.87) | 41 (28.67) |  |
| Tumor size, No. (%), cm |  |  |  |  |  |  |
| <=3 | 23 (6.57) | 58 (12.53) | 0.016 | 7 (4.90) | 15 (10.49) | 0.206 |
| (3-5) | 159 (45.43) | 206 (44.49) |  | 63 (44.06) | 60 (41.96) |  |
| >=5 | 168 (48.00) | 199 (42.98) |  | 73 (51.05) | 68 (47.55) |  |
| Vascular invasion, No. (%) |  |  |  |  |  |  |
| No | 289 (89.20) | 275 (70.51) | <0.001 |  |  |  |
| Yes | 35 (10.80) | 115 (29.49) |  |  |  |  |
| Perineural invasion, No. (%) |  |  |  |  |  |  |
| No | 248 (81.31) | 225 (59.84) | <0.001 |  |  |  |
| Yes | 57 (18.69) | 151 (40.16) |  |  |  |  |
| CEA, No. (%) |  |  |  |  |  |  |
| Negative | 235 (67.14) | 305 (65.87) | 0.761 | 92 (64.34) | 87 (60.84) | 0.625 |
| Positive | 115 (32.86) | 158 (34.13) |  | 51 (35.66) | 56 (39.16) |  |
| CA199, No. (%) |  |  |  |  |  |  |
| Negative | 223 (63.71) | 316 (68.25) | 0.201 | 102 (71.33) | 94 (65.73) | 0.373 |
| Positive | 127 (36.29) | 147 (31.75) |  | 41 (28.67) | 49 (34.27) |  |
| Tumor grade, No. (%) |  |  |  |  |  |  |
| Grade (1/2 ) | 274 (78.29) | 346 (74.73) | 0.273 | 114 (79.72) | 105 (73.43) | 0.264 |
| Grade (3/4) | 76 (21.71) | 117 (25.27) |  | 29 (20.28) | 38 (26.57) |  |
| Tumor pathological T stage, No. (%) |  |  |  |  |  |  |
| T1/2_stage | 6 (1.71) | 263 (56.80) | <0.001 | 6 (4.20) | 6 (4.20) | 1 |
| T3/4_stage | 344 (98.29) | 200 (43.20) |  | 137 (95.80) | 137 (95.80) |  |
| Tumor pathological N stage, No. (%) |  |  |  |  |  |  |
| N0_stage | 326 (93.14) | 321 (69.33) | <0.001 | 120 (83.92) | 120 (83.92) | 1 |
| N1/2_stage | 24 (6.86) | 142 (30.67) |  | 23 (16.08) | 23 (16.08) |  |
| No. of harvested lymph nodes, No. (%) |  |  |  |  |  |  |
| >=12 | 332 (94.86) | 440 (95.03) | 1 | 140 (97.90) | 139 (97.20) | 1 |
| <12 | 18 (5.14) | 23 (4.97) |  | 3 (2.10) | 4 (2.80) |  |

Abbreviation: CEA, carcinoembryonic antigen; CA199, Carbohydrate antigen 199.
